# Urinary Exosomal miRNA Signature in Type II Diabetic Nephropathy Patients

**DOI:** 10.1371/journal.pone.0150154

**Published:** 2016-03-01

**Authors:** Denis Delić, Claudia Eisele, Ramona Schmid, Patrick Baum, Franziska Wiech, Martin Gerl, Heike Zimdahl, Steven S. Pullen, Richard Urquhart

**Affiliations:** 1 Translational Medicine & Clinical Pharmacology, Boehringer Ingelheim Pharma GmbH & Co. KG, Birkendorferstr.65, 88397 Biberach, Germany; 2 R&D Project Management, Boehringer Ingelheim Pharma GmbH & Co. KG, Birkendorferstr.65, 88397 Biberach, Germany; 3 Cardiometabolic Diseases Research, Boehringer Ingelheim Pharmaceuticals Inc., 900 Ridgebury Road, Ridgefield, Connecticut 06877–0368, United States of America; 4 Translational Medicine & Clinical Pharmacology, Boehringer Ingelheim Pharmaceuticals Inc., 900 Ridgebury Road, Ridgefield, Connecticut 06877–0368, United States of America; National University of Singapore, SINGAPORE

## Abstract

MicroRNAs (miRNAs) are short non-coding RNA species which are important post-transcriptional regulators of gene expression and play an important role in the pathogenesis of diabetic nephropathy. miRNAs are present in urine in a remarkably stable form packaged in extracellular vesicles, predominantly exosomes. In the present study, urinary exosomal miRNA profiling was conducted in urinary exosomes obtained from 8 healthy controls (C), 8 patients with type II diabetes (T2D) and 8 patients with type II diabetic nephropathy (DN) using Agilent´s miRNA microarrays. In total, the expression of 16 miRNA species was deregulated (>2-fold) in DN patients compared to healthy donors and T2D patients: the expression of 14 miRNAs (miR-320c, miR-6068, miR-1234-5p, miR-6133, miR-4270, miR-4739, miR-371b-5p, miR-638, miR-572, miR-1227-5p, miR-6126, miR-1915-5p, miR-4778-5p and miR-2861) was up-regulated whereas the expression of 2 miRNAs (miR-30d-5p and miR-30e-5p) was down-regulated. Most of the deregulated miRNAs are involved in progression of renal diseases. Deregulation of urinary exosomal miRNAs occurred in micro-albuminuric DN patients but not in normo-albuminuric DN patients. We used qRT-PCR based analysis of the most strongly up-regulated miRNAs in urinary exosomes from DN patients, miRNAs miR-320c and miR-6068. The correlation of miRNA expression and micro-albuminuria levels could be replicated in a confirmation cohort. In conclusion, urinary exosomal miRNA content is altered in type II diabetic patients with DN. Deregulated miR-320c, which might have an impact on the TGF-β-signaling pathway via targeting thrombospondin 1 (TSP-1) shows promise as a novel candidate marker for disease progression in type II DN that should be evaluated in future studies.

## Introduction

Diabetic nephropathy (DN), the most frequent form of chronic kidney disease, is a progressive kidney disease and a major debilitating complication of both type 1 and type 2 diabetes that can lead to end-stage renal disease (ESRD) [1]. Albuminuria is widely used as a biomarker for DN. However, clinical relevance as a surrogate end-point in chronic kidney disease is controversial [2–4] and recent studies suggest that micro-albuminuria is a less precise predictor of DN risk [5,6]. Therefore, there is an unmet need to identify biomarkers reflecting early effects during disease development and progression and which potentially allow monitoring predictive and/or prognostic treatment effects.

MicroRNAs (miRNAs) are small, 18–22 nucleotides in length, non-coding RNA molecules that modulate differentiation, growth, apoptosis and proliferation of cells by interfering with protein synthesis by either inducing mRNA degradation or repressing translation [7–9]. The expression of miRNAs-192, -194, -204, -215 and -216 is increased in kidney compared to other tissues [10]. There is strong evidence that miRNA-dependent mechanisms for TGF-β-induced expression of extracellular matrix (ECM) genes are implicated in the pathogenesis of DN [11]. In particular, miRNA-192 has been shown to be induced in glomerular mesangial cells in experimental diabetic nephropathy where it promotes collagen production [12] and mediates TGF-β/Smad3-induced tubulo-interstitial fibrosis [11]. Moreover, the miRNA-30 family is abundantly expressed in podocytes compared to other glomerular cell types [13] and its Smad2-dependent down-regulation [14] facilitates podocyte injury [15]. Glomerular miR-21 expression is positively associated with albumin-creatinine ratio (ACR) in patients with DN and potentially serves as an indicator of podocyte damage [16]. Increased circulating miR-21 levels are associated with kidney fibrosis and correlate with eGFR [17]. Recently, it was demonstrated that renal miR-21 levels are positively correlated with estimated glomerular filtration rate (eGFR), proteinuria and glomerulosclerosis in DN patients [18]. Furthermore, the miRNA-29 family protects the kidney from fibrotic damage and the DPP-4 inhibitor linagliptin has been shown to inhibit TGF-β-induced endothelial to mesenchymal transition (EndMT) by restoring miRNA-29s level [19].

Cell-free circulating miRNAs are present in various body fluids, such as serum, plasma and urine, and in DN may reflect responses to various pathophysiological stresses [20,21]. Urine is an ideal source of biomarkers for renal diseases and several studies have indicated miRNAs as potential biomarkers. Changes in cell-free urinary miRNAs in type 1 diabetes mellitus patients have been reported to be associated with different stages of albuminuria and nephropathy [22]. Micro-albuminuria was associated with decreased levels of miRNA-323b-5p and increased urine concentration of miRNA-429 in patients with long-lasting type I diabetes [22]. Moreover, a miRNA signature was identified that has the potential to predict the development of micro-albuminuria in patients with type I diabetes [23].

Urine contains various types of extracellular vesicles, exosomes being the most-well characterized so far. Exosomes are cup-shaped 40–100 nm membrane vesicles that derive from the multivesicular bodies in the endocytic compartment [24]. Exosomal cargo, including miRNAs can be delivered to distant target cells, which is increasingly being recognized as an important mode of cell-cell communication [24]; in addition, a potential source of novel non-invasive biomarkers for renal diseases [25]. In contrast to free urinary miRNAs, exosomal miRNAs are remarkably stable as they are protected from endogenous RNase activity. In particular, free urinary miRNAs are not the optimal source for biomarker discovery in renal pathophysiology as they are confounded by plasma miRNAs which are able to pass the glomerular filtration barrier due to their small molecular weight [26]. Exosome size is over the cut-off value for glomerular filtration even in patients with macroalbuminuria. A recent study showed that exosomes can cross the brain blood barrier [27], however presumably via receptor mediated transcytosis [28]. It is unknown, whether this mechanism is important in the kidney. Nevertheless, there is ample evidence that large quantities of exosomes are secreted from all nephron segments [29,30] and may thus provide valuable insights in renal pathophysiology. Recent studies revealed that urinary exosomal miRNA content is changed in patients with focal segmental glomerulosclerosis [31] and in type I diabetic patients with incipient diabetic nephropathy [32]. Moreover, miR-29c level in urinary exosomes was shown to potentially serve as predictor of early fibrosis in lupus nephritis [33].

In the present study, we identified an altered miRNA signature in urinary exosomes from type II DN patients for the first time. This deregulation of distinct urinary exosomal miRNA species correlates with degree of micro-albuminuria. Notably, the enriched expression of miRNA-320c which is indirectly involved in TGF-β signaling via targeting TSP-1 may represent a novel candidate marker for early progression of disease and/or early treatment effects.

## Materials and Methods

### Patient characteristics

The patients in this study were defined as being healthy (no diabetes and estimated glomerular filtration rate [eGFR] >90; no historical eGFR available), diabetic (historical eGFR >90), or with diabetic nephropathy (historical eGFR <60). Historical eGFR was based on information in medical records collected prior to the date of urine collection for each subject. Clinical and laboratory characteristics of the screening cohort consisting of 8 healthy controls (C), 8 patients with type II diabetes (T2D) and 8 subjects with type II diabetic nephropathy (DN) are summarized in Table 1.

**Table 1:** Clinical and Laboratory parameters of healthy controls, T2D and DN patients from the screening cohort.

	Controls	T2D	DN
N	8	8	8
Age (years)	30.9 ± 7.9	56.2 ± 15	67.1 ± 7.8
Sex (female/male)	4/4	3/5	4/4
Historical eGFR [ml/min/1.73m^2^]	—	101.25 ± 7.45	35.25 ± 2.65*
UACR [mg/g]	0.69 ± 0.23	1.11 ± 0.32	93.52 ± 37.72*

eGFR was calculated using the Modification of Diet in Renal Disease Study equation (Levey et al., 1999)

*Significant differences against controls and T2D group are indicated by * (*P*<0.05).

Among the 8 type II diabetic nephropathy patients are 5 with micro-albuminuria (ACR of 30–300 mg albumin/g creatinine) and 3 with normo-albuminuria (ACR of < 30 mg albumin/g creatinine). The second confirmation cohort consists of 6 healthy subjects, 6 patients with type II diabetes and 5 micro-albuminuric type II diabetic nephropathy patients (Table 2).

**Table 2:** Clinical and Laboratory parameters of healthy controls, T2D and DN patients from the confirmation cohort.

	Controls	T2D	DN
N	6	6	5
Age (years)	39 ± 19.9	64.8 ± 12.1	72 ± 10.1
Sex (female/male)	5/1	4/2	3/2
Historical eGFR [ml/min/1.73m^2^]	—	95.2 ± 5.02	45.4 ± 13.5*
UACR [mg/g]	0.5 ± 0.13	1.95 ± 0.73	99.7 ± 49.9*

eGFR was calculated using the Modification of Diet in Renal Disease Study equation (Levey et al., 1999)

*Significant differences against controls and T2D group are indicated by * (*P*<0.05).

All relevant medication information is summarized in S1 Table. Urine samples were obtained from Bioreclamation IVT (NY, USA). Processing involved centrifugation at 2000 g for 10 min and the supernatant was removed and stored at -80°C until use.

### Urinary exosomal miRNA isolation

Exosomes were isolated from 3 ml urine using the exosome precipitation reagent ExoQuick-TC (System Biosciences; Mountain View, CA). The modified exosome precipitation protocol from Alvarez et al. [34,35] was used. Samples were centrifuged for 15 min at 3,000 x g and 1/3 volume ExoQuick TC (System Biosciences) was added to the supernatant. After overnight precipitation at 4°C samples were centrifuged for 30 min at 10,000 g at room temperature and the resulting exosome pellet was resuspended with 200μl exosome buffer and subsequently lysed for 5 min with 150μl lysis buffer at RT. Exosomal miRNAs were isolated using SeraMir Exosome RNA Amplification Kit (System Biosciences) according to manufacturer´s protocol. In the independent confirmation cohort urinary exosomal miRNAs were isolated using the exoRNeasy Serum/Plasma Maxi Kit (QIAGEN, Hilden, Germany) according to manufacturer´s instructions. Quality control was performed on the Agilent 2100 Bioanalyzer, using the RNA 6000 Nano Kit (Agilent Technologies Inc, Waldbronn, Germany) and miRNA concentration was measured using Quant-IT micro RNA assay kit (Life Technologies) according to manufacturer´s instructions.

### miRNA microarray

Samples were analyzed with the High-Resolution Microarray Scanner GS2505_C (Agilent Technologies, Waldbronn, Germany) using the CBC (Comprehensive Biomarker Center, Heidelberg, Germany) custom content Agilent microarray slides which contain all microRNAs from the Sanger miRBase release 19 [36]. Each glass slide is formatted with 8 high-definition 60K arrays (8x60K design/8arrays with 60,000 features each). In addition to 20 replicates of each microRNA, each array carries control probes for grid alignment as well as labeling and hybridization of control spike-ins. Agilent´s microRNA complete labeling and hybridization kit (Agilent Technologies) contains Cyanine 3-Cytidine biphosphate (pCp) for labeling and the hybridization time was 20 hours at 55°C with 20 rpm. Results were analyzed using the Agilent Feature Extraction software (10.7.3.1) (Agilent Technologies).

### Data analysis

Data was analyzed using R version 3.0.1 [37] in conjunction with Bioconductor version 2.13 [38]. For background correction and normalization we utilized the rmaMicroRna-function available in the AgiMicroRna version 2.12.0 Bioconductor package [39]. In brief, rmaMicroRna obtains background corrected intensity by fitting a normal + exponential convolution model, quantile normalizes intensities between arrays and log_2_ transforms the probe intensities. Finally, probe sets that interrogate the same microRNA are summarized by fitting a linear model to the log_2_ transformed intensities. For outlier identification all correlation coefficients were transformed with the Fisher transformation and then applied to the robust test according to Hampel´s rule [40].

To assess differential expression, linear models were fitted to the data and empirical Bayes moderation was applied to compute moderated t-statistics using limma version 3.18.13 [41]. miRNAs with at least two-fold deregulation and a p-value <0.05 in any group-wise comparison were considered differentially expressed. *P* values were corrected using Benjamini-Hochberg method for multiple testing [42,43]. miRNA expression profiles between individual samples were overall compared by principal component analysis. Data are publicly available at the NCBI Gene Expression Omnibus repository (Array Express accession number: A-MTAB-579).

To visualize the differentially expressed miRNAs, a heat map was generated in which each value was normalized to the mean expression value of the control group (Spotfire, TIBCO Software Inc., Palo Alto, United States). To predict target genes and related pathways of each deregulated miRNA miRWalk database was used (http://www.umm.uni-heidelberg.de/apps/zmf/mirwalk). Potential influence on biological pathways was performed by Ingenuity Pathway Analysis (QIAGEN, Redwood City, United States).

### Quantitative real-time PCR

miRNAs were reverse transcribed using TaqMan® microRNA Reverse Transcription Kit (Life Technologies) and TaqMan miRNA assays (Life Technologies) specific for miR-320c (assay ID: 241053_mat), miR-6078 (470895_mat), miR-6076 (474248_mat) and miR-197-5p (474626_mat). TaqMan® gene expression master mix (Life Technologies) was used for the PCR reaction, which was performed according manufacturer´s protocol on a 7900HT real-time PCR System. All samples were run in duplicates and raw ct values were calculated using the SDS software v.2.4. miR-197-5p was determined as a suitable candidate for normalization using NormFinder software [44]. Fold-change of expression was calculated using the comparative Ct method (2^-ΔΔct^) [45].

### Statistical analysis of real-time PCR

All bars shown represent mean +/- standard error. Data sets were analyzed for statistical significance using two-tailed unpaired Student´s t-test (* = p<0.05).

## Results

### Expression analysis

The aim of this was to identify miRNA species in urinary exosomes from patients with DN which significantly differ in their expression profile compared to healthy controls and to patients with T2D. To this end miRNAs were isolated from urinary exosomes from 8 healthy controls (C), 8 patients with T2D and 8 patients with DN and subjected to Agilent microarrays containing all miRNAs from miRBase release 19.0. Quantitative real-time PCR was used to verify expression profiles and to confirm the results from the screening cohort in the independent confirmation cohort.

Deregulated miRNAs were identified by moderated t-test evaluating only those miRNAs with at least two-fold expression changes at a *P*-value < 0.05. In addition, *P*-values were further corrected with Benjamini-Hochberg method based multiple testing. The biological significance of selected miRNAs was further established with qRT-PCR. Functional annotations of these miRNAs were searched in several databases including miRWalk (http://www.umm.uni-heidelberg.de/apps/zmf/mirwalk), TargetScan (http://www.targetscan.org), miRBase (http://www.mirbase.org), and PubMed (http://www.ncbi.nlm.nih.gov/pubmed).

### Differentially regulated miRNA-signature in urinary exosomes from type II DN patients

**Fig 1:**
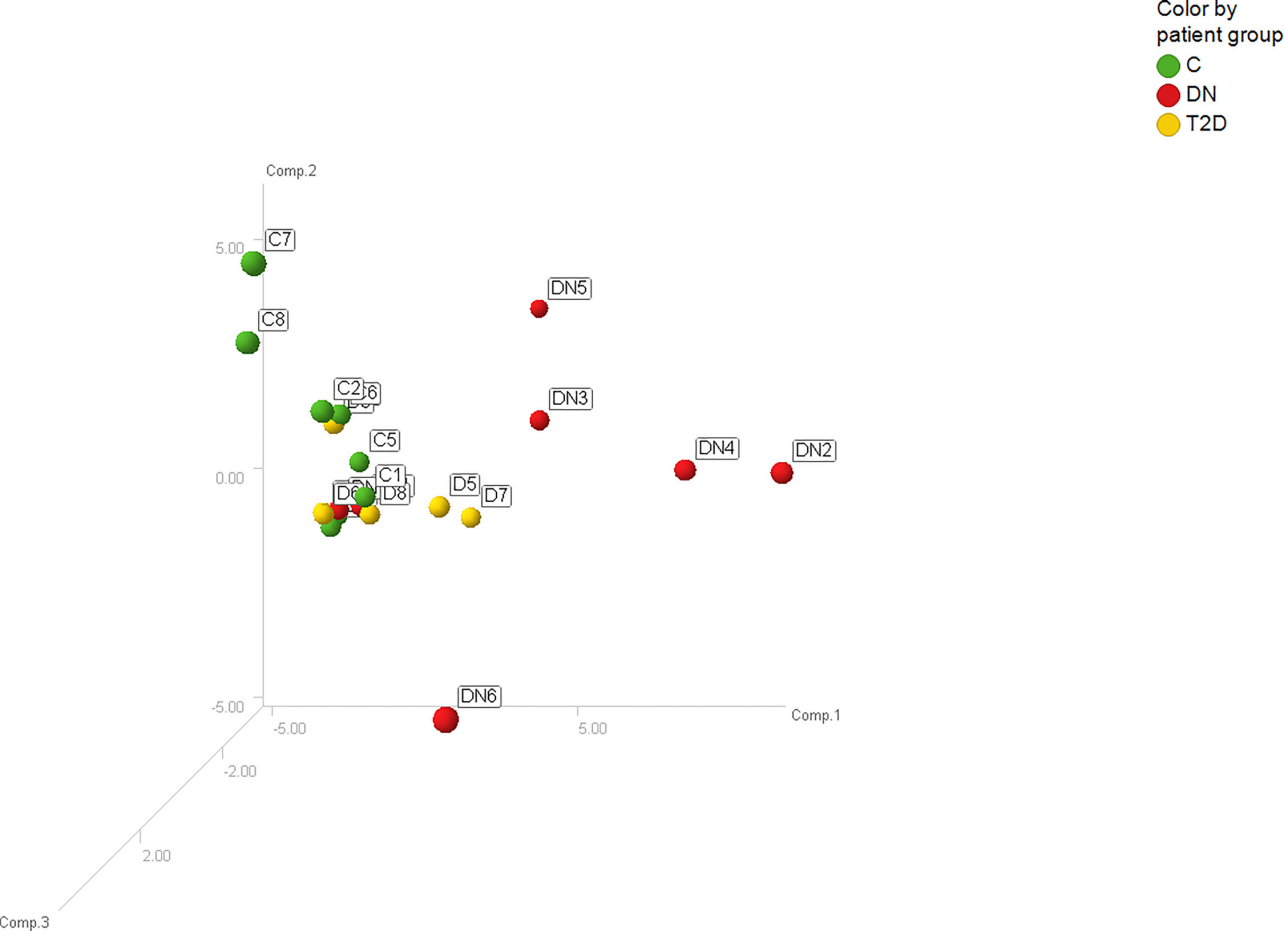
Principal component analysis. Displayed are the first three major components from the principal component analysis. (green = C; yellow = T2D; red = DN).

**Fig 2:**
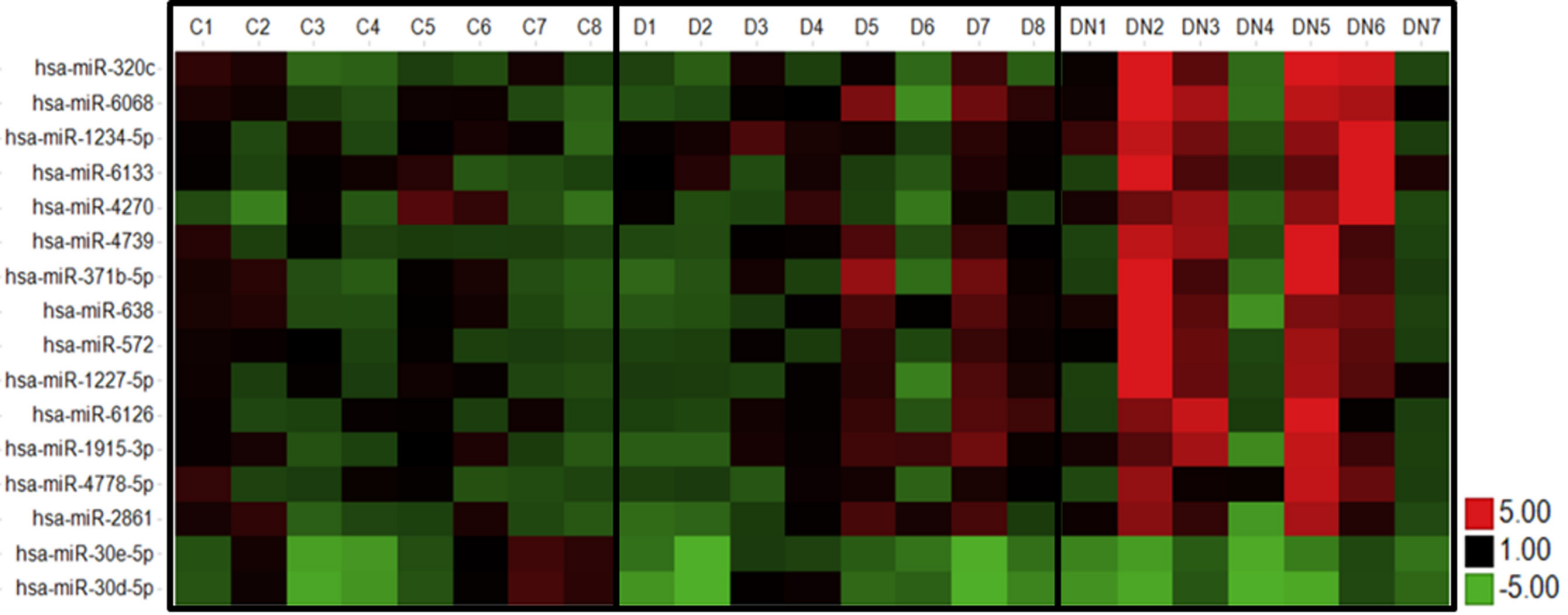
Heat map of expression levels of differentially expressed miRNAs in DN patients. Fold change of expression levels were normalized to the mean signal intensities of healthy controls. Red and green colors represent fold change up- and down-regulation, respectively, as indicated by the linear scale bar (C = healthy controls; D = T2D patients; DN = type II diabetic nephropathy patients).

Principal component analysis revealed that the expression profiles were relatively similar among the eight healthy controls and T2D patients indicating low inter-individual variability (Fig 1). One outlier was identified among the 8 DN patients so that only seven patient samples were further analyzed. In total 309 miRNAs were detected in urinary exosomes. Both, the healthy control and the T2D groups, differed from DN2, DN3, DN4, DN5 and DN6. None of the detected miRNA species was deregulated in urinary exosomes from T2D patients compared to healthy controls. Overall the expression of 14 urinary exosomal miRNAs was up-regulated at least 2-fold whereas the expression of 2 miRNA species was down-regulated at least 2-fold in DN patients compared to healthy subjects and T2D (Fig 2). The functions of the deregulated miRNAs based on published data and their experimentally observed and/or predicted target mRNAs are summarized in Table 3.

**Table 3:** Differentially regulated miRNAs in DN patients.

miRNA	DN/C	raw *P*	adj. *P*	DN/T2D	raw *P*	adj. *P*	Function (sample matrix)	Target mRNA	PMID
miR-320c	4.83	<0.01	0.08	5.01	<0.01	0.16	Up-regulated in renal cortex of lupus nephritis patients (tissue).	THBS1, BMP1	18998140
miR-6068	3.85	<0.01	0.08	2.61	<0.01	0.41	—	—	—
miR-1234-5p	3.6	<0.01	0.08	2.5	0.02	0.51	Up-regulated in patients with minimal change disease (urine).	STAT3	25682967
miR-6133	2.95	<0.01	0.08	2.66	<0.01	0.41	—	SMAD7	
miR-4270	2.87	0.02	0.36	2.97	0.04	0.59	Down-regulated in patients with focal segmental glomerulosclerosis (plasma).	NR1D2, LAMTOR	25682967
miR-4739	2.41	0.02	0.37	1.72	0.05	0.66	Induced expression in gastric cancer after interference with β-catenin (FFPE).	CTNNB1	25861021
miR-371b-5p	2.7	<0.01	0.32	1.93	0.02	0.52	Up-regulated in patients with lupus nephritis (PBMCs).	IRF5, TRIAP1	20485490
miR-638	2.71	<0.01	0.08	2.07	<0.01	0.41	Up-regulated in patients with lupus nephritis (PBMCs).	PGK1	20485490
miR-572	2.57	<0.01	0.08	2.14	<0.01	0.41	Increased in early state renal cell carcinoma (serum).	SOCS1, THBS1	25556603
miR-1227-5p	2.51	<0.01	0.24	2.06	0.01	0.41	Up-regulated in oncosomes produced by prostate cells (RWPE2 cells).	—	24091630
miR-6126	2.41	0.02	0.35	1.72	0.09	0.81	—	—	—
miR-1915-5p	2.25	0.03	0.44	1.51	0.18	0.93	Up-regulated in patients with minimal change disease (plasma).	TLR2	25682967
miR-4778-5p	2.17	0.01	0.35	2.21	0.01	0.41	—	STAT3	—
miR-2861	2.13	0.02	0.37	2.69	0.06	0.68	Up-regulated expression in papillary tyroid carcinoma with lymph node metastasis (tissue).	VEGF-A, FOXO1	23609190
miR-30e-5p	0.36	0.04	0.47	0.79	0.81	0.99	Down-regulation of miR-30 family is required for TGF-β induced apoptosis in podoytes.	PTPN13, FRMD6	24086574
miR-30d-5p	0.34	0.04	0.47	0.7	0.8	0.99	Down-regulation of miR-30 family is required for TGF-β induced apoptosis in podoytes.	STAT3, TNF	24086574

miR-320c showed the strongest up-regulation (5-fold). The 9 miRNA species miR-320c, miR-1234-5p, miR-4270, miR-371b-5p, miR-638, miR-572, miR-1915-5p, miR-30d-5p and miR-30e-5p are involved in renal diseases, such as focal segmental glomerulosclerosis, minimal change disease, renal cell carcinoma and lupus nephritis (Table 3). miR-4739, miR-1227-5p and miR-2861 are involved in cancerous diseases, such as gastric cancer (Table 3). Moreover, miR-6068, miR-6133, miR-6126 and miR-4778-5p have unknown functions to date (Table 3).

**Fig 3:**
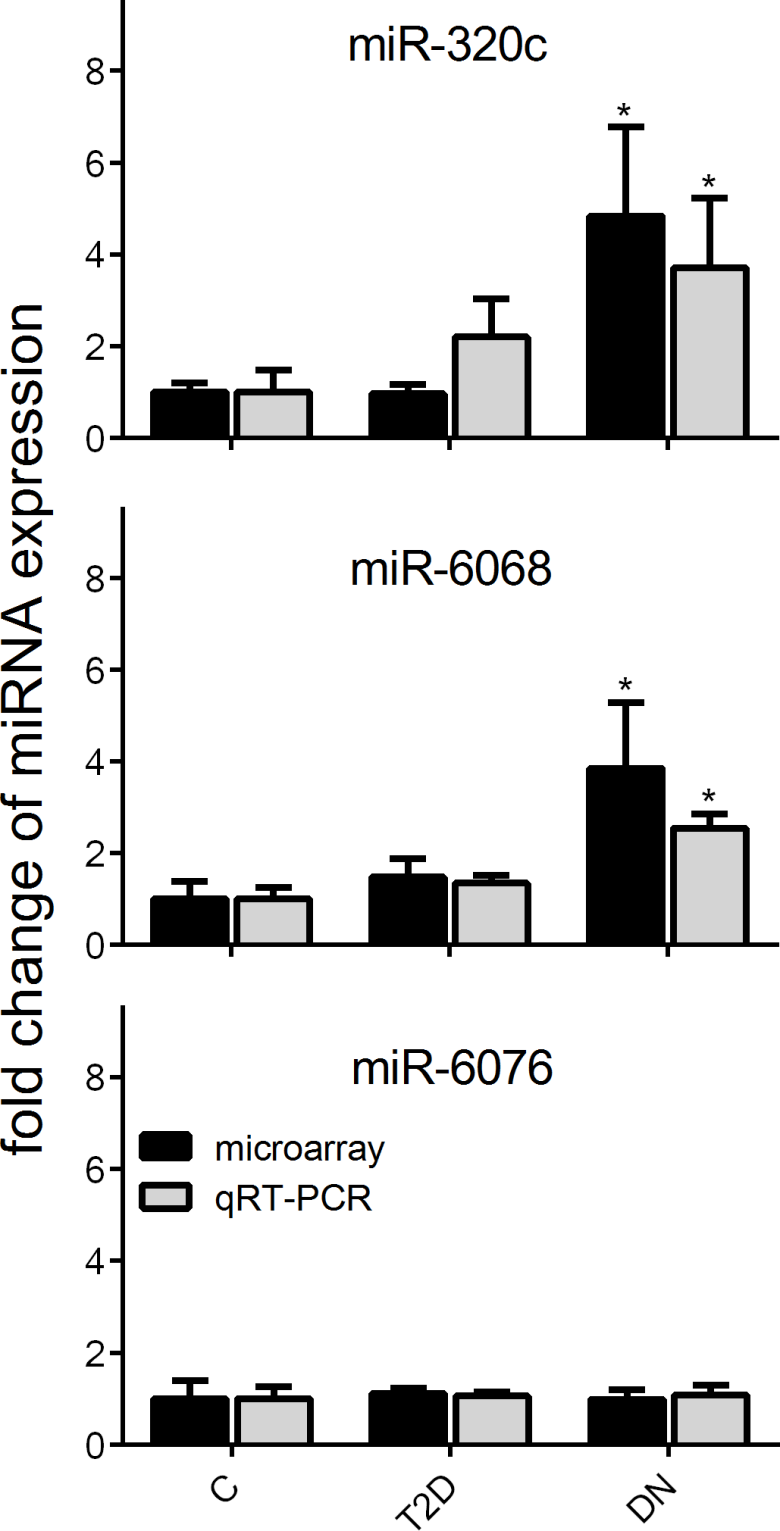
Expression of differentially expressed miRNAs in DN patients analyzed by microarray and qRT-PCR. Gene expression profiles of up-regulated miR-320c and miR-6068 and unresponsive miR-6076 were verified by qRT-PCR. Relative miRNA expression was normalized to the mean expression of healthy controls. Significant differences to controls are indicated by * (*P*<0.05).

The miRNA species miR-320c and miR-6068 that were most strongly up-regulated in urinary exosomes from DN patients were chosen for confirmation of results by qRT-PCR in all subjects. miR-6076 served as negative control whose expression level is constant across all subjects. Consistent with profiling results qRT-PCR analysis showed that the expression levels of urinary exosomal miR-320c and miR-6068 were significantly higher in DN patients whereas the expression of miR-6076 was not influenced (Fig 3).

### Replication of results in the confirmation cohort

**Fig 4:**
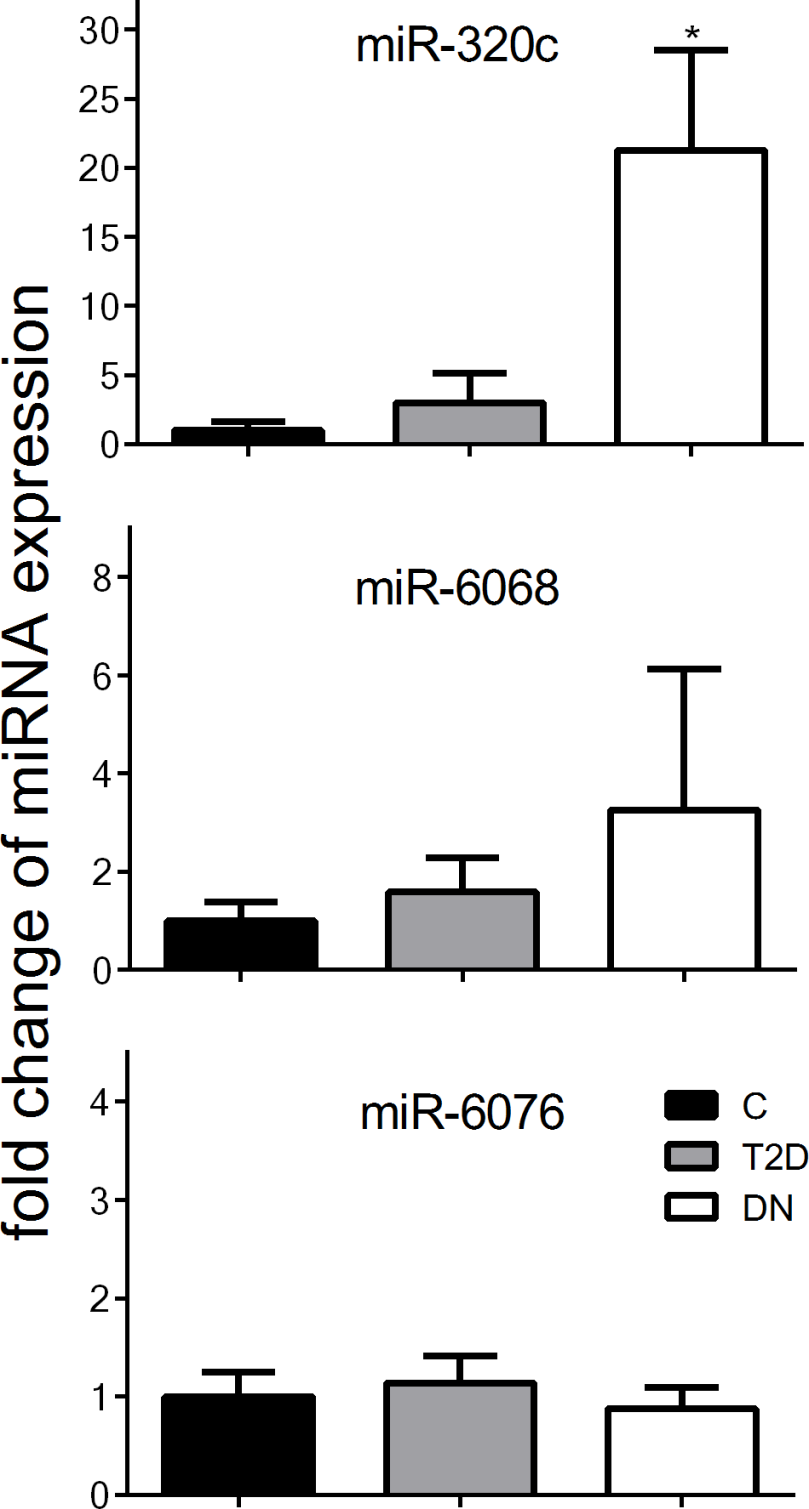
Expression of differentially expressed miRNAs in the confirmation cohort. Expression of differentially expressed miRNAs miR-320c and miR-6068 and unresponsive miR-6076 in a second cohort were verified by qRT-PCR. Relative miRNA expression was normalized to the mean expression of healthy controls. Significant differences to controls are indicated by * (*P*<0.05).

A second independent cohort consisting of 6 healthy controls, 6 T2D patients and 5 with DN was used to confirm the results from the screening cohort. The urinary exosomal expression levels of miR-320c, miR-6068, and miR-6076 were analyzed by qRT-PCR. To show the independence of the extraction method, an alternative exosomal miRNA isolation method was implicated using ExoRNeasy Kit which does not need a time-consuming overnight precipitation. Remarkably, the expression of miR-320c was significantly induced in urinary exosomes from DN patients compared to healthy controls and T2D patients, whereas the expression of miR-6076 was unaffected (Fig 4).

### Correlation of up-regulation of miRNA-320c expression and micro-albuminuria

**Fig 5:**
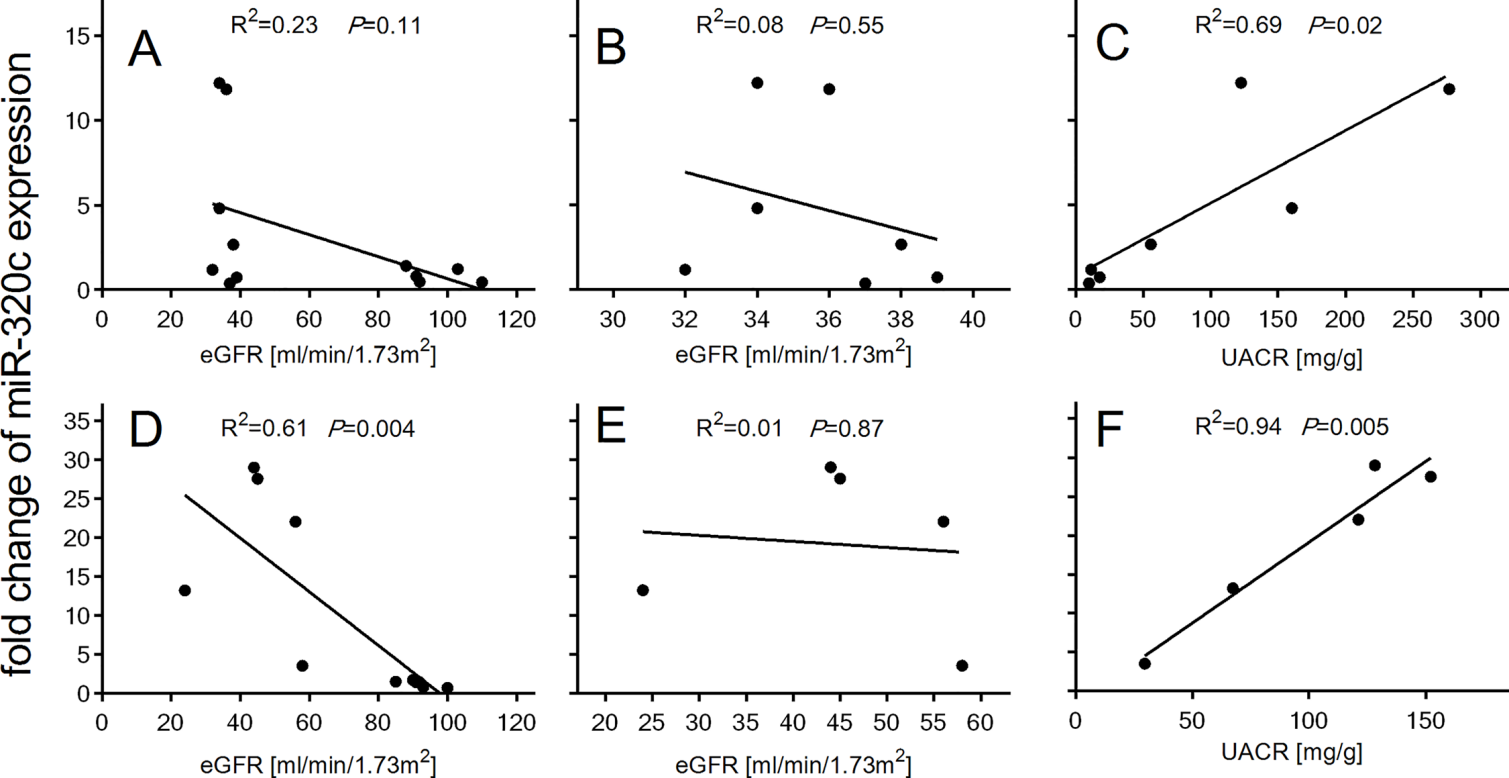
miR-320c expression correlation with eGFR and UACR. Correlation between miR-320c expression in T2D and DN patients and historical eGFR in the screening cohort (A) and the confirmation cohort (D). Correlation between miR-320c expression only in DN patients and historical eGFR in the screening cohort (B) and the confirmation cohort (E). Correlation between miR-320c expression in DN patients and UACR in the screening cohort (C) and the confirmation cohort (F). Data were compared by Pearson´s correlation coefficient.

Correlation analyses revealed that up-regulated urinary exosomal miR-320c expression negatively correlated with increasing eGFR (screening cohort: R^2^ = 0.23, *P* = 0.11; confirmation cohort: R^2^ = 0.61, *P* = 0.004) (Fig 5A and 5D). Urinary exosomal miR-320c levels in DN patients did not significantly correlate with eGFR in either cohorts (screening cohort: R^2^ = 0.08, *P* = 0.55; confirmation cohort: R^2^ = 0.01, *P* = 0.87) (Fig 5B and 5E), but significantly positively correlated with increasing UACR (screening cohort: R^2^ = 0.69, *P* = 0.02; confirmation cohort: R^2^ = 0.94, *P* = 0.005) (Fig 5C and 5F). Among the 7 DN patients, 3 were normo-albuminuric and 4 were micro-albuminuric. The expression of miR-320c was strongly up-regulated in subjects with micro-albuminuria whereas the expression was unaffected in subjects with normo-albuminuria (Fig 5C). Furthermore, the 4 subjects with micro-albuminuria can also be identified in the principal component analysis (Fig 1) and the heat map (Fig 2) as indicated by the highest deregulation in patients DN2, DN3, DN5 and DN6. The 5 DN patients from the confirmation cohort were all micro-albuminuric.

## Discussion

This study provides first evidence that the miRNA profile is altered in urinary exosomes from type II DN patients. Expression profiling of urinary exosomal miRNA revealed a differential miRNA signature in type II DN patients: 14 miRNA species were up-regulated and 2 miRNA-species were down-regulated at least 2-fold. Principal component analysis and the heat map of deregulated miRNAs revealed that differential urinary exosomal miRNA expression occurs in patients with micro-albuminuria compared to normo-albuminuric patients. This was further substantiated in the confirmation cohort where all patients had micro-albuminuria. Urinary exosomal miRNA content is altered in T1D patients with incipient diabetic nephropathy and micro-albuminuria resulting in an up-regulation of miR-130a and miR-145 and a down-regulation of miR-155 and miR-424 [32]. These miRNA species are known to influence the TGF-β signaling pathway [46,47]. Our results did not reveal a deregulation of these miRNAs. It is noteworthy to mention that in the study of Barutta et al. [32] patients had T1D and a very low degree of micro-albuminuria and normal renal function whereas our patients had stage 3 and 4 DN.

Most of our identified urinary exosomal miRNAs are known to be deregulated in various renal diseases. The expression of miR-1234-5p and miR-371-5p is up-regulated in plasma samples from patients with minimal change disease (MCD) and the urinary expression of miR-1915-5p is up-regulated in MCD patients [31]. miR-1915-5p is involved in the regulation of adult renal progenitor cells (ARPCs) via regulation of renal stem cell markers such as CD133 and PAX2 [48]. Moreover, TLR2 as an important marker of acute tubular cell injury is regulated by miR-1915-5p by driving ARPC to repair damaged renal proximal tubular epithelial cells by secretion of inhibin-A, decorin and cyclinD1 [49]. Up-regulation of miR-1915-5p expression would lead to an inhibition of important renal repair mechanisms. Another important aspect of renal injury is the SMAD2-dependent down-regulation of miR-30 which is required for TGF-β-induced apoptosis of podocytes in glomerulosclerosis [14]. Furthermore, down-regulation of miR-30 expression is prevented by glucocorticoid treatment of podocytopathy [15]. Increased expression of miR-572 was also observed during early-stage renal cell carcinoma (RCC) and is part as a potential diagnostic tool for early-stage RCC [50].

Recently, several studies have explored the potential of urinary miRNA expression levels in DN patients as biomarkers of disease progression. Renal fibrosis is a hallmark of various chronic kidney diseases and TGF-β is the key mediator of renal fibrosis [51]. Increased mesangial expression of TGF-β-induced miRNA-192, one of the most well studied miRNAs in DN, leads to increased renal fibrosis [12]. Neither free urinary miRNA profiling in type I DN patients [22] nor urinary exosomal miRNA profiling in incipient DN patients [32] revealed up-regulation of miR-192 expression. In contrast to the above findings with free urinary miRNA, our profiling of urinary exosomes revealed undetectable miR-192 levels consistent with the concept that exosomes predominantly carry miRNAs that are delivered to distant target cells. Conversely, members of both the miR-29 and miR-200 families are negatively regulated by TGF-β, and protect against renal fibrosis by inhibiting epithelial-to-mesenchymal transition and preventing the deposition of extracellular matrix, respectively [52–54]. Moreover, miR-29 levels in urinary exosomes were significantly down-regulated in chronic kidney disease patients [55] and in patients with lupus nephritis [33]. In mice, induction of DPP-4 in diabetic kidneys was associated with suppressed levels of miR-29 members. Interestingly, treatment with the DPP-4 inhibitor linagliptin leads to restored miR-29 levels and associated suppressed DPP-4 protein levels [16]. Our study also revealed suppressed miR-29 and miR-200 levels in urinary exosomes from T2D patients and this effect was further pronounced in type II DN patients, but this was not significant (data not shown) and therefore not included in our candidate list.

Patients with DN included in both cohorts received standard of care medication, such as angiotensin converting enzyme inhibitors (ACEi) and/or angiotensin receptor blockers (ARBs). In general, effects of ACEi and ARBs on miRNA expression are described for various cardiovascular diseases, such as coronary artery disease (CAD), and combined treatment of ACEi and statin decreased miR-146a/b levels in peripheral blood mononuclear cells from CAD patients [56]. Treatment with ACE inhibitors results in down-regulation of miR-324-3p expression in kidney tissue [57,58]. miR-324-3p is the most up-regulated miRNA species in kidney biopsies from Munich Wistar Fromter rats which develop spontaneous progressive nephropathy [57]. Prolyl endopeptidase, a serine peptidase involved in the metabolism of angiotensins, is a prominent target of miR-324-3p and its expression is markedly decreased. Our data revealed no significant deregulation of miR-324-3p in urinary exosomes (FC: 0.88, *P* = 0.84). Treatment with ACEi/ARBs led to decreased up-regulation of renal miR-21 levels in DN patients [18]. Our data set showed 1.8-fold up-regulated miR-21 levels in DN patients (*P* = 0.3). To date, there is no data available concerning the influence of ACEi/ARBs on urinary exosomal miRNA expression.

Our study revealed a strong up-regulation of miR-320c in urinary exosomes from type II DN patients. Interestingly, let-7d, miR-203 and miR-320 were identified with two other miRNA profiling platforms including next-generation sequencing and qPCR-based TaqMan low density arrays as novel urinary biomarkers for drug induced renal tubular epithelial injury in rats [59]. miR-320c regulates human cartilage metabolism by targeting ADAMTS5 which is an efficient aggrecanase and induces cartilage degeneration [60]. Moreover, miR-320c is involved in the regulation of adipocytic differentiation of human mesenchymal skeletal stem cells [61] and inhibits tumorous behaviors of bladder cancer by targeting CDK6 [62]. miR-Walk database [63] and Ingenuity pathway analysis (IPA) showed thrompospondin 1 (TSP-1), TSP-4 and bone morphogenetic 6 (BMP6) as predicted targets both of which are involved in the regulation of TGF-β signaling. BMP6 is able to ameliorate TGF-β induced changes in HK-2 cells [64]. Thus, up-regulated miR-320c expression could induce down-regulation of BMP6 and aggravate renal fibrosis. TSP-1 is a major activator of TGF-β in fibrotic renal disease [65,66] and TSP-1 protein is increased in the glomeruli of patients with types 1 and 2 DN, which correlates with TGF-β activity [67,68]. Moreover, in a murine model of DN it was shown that a blockade of TSP-1 dependent TGF-β activity reduces renal injury and proteinuria [69]. TSP-1 is a predicted direct target of miR-320c and thus potentially negatively influences the TGF-β pathway (S1 Fig) as a compensatory consequence of the up-regulated TGF-β signaling pathway. TSP-4 is an important regulator of tissue remodeling via regulating collagen synthesis and TSP-4 deficiency markedly increases cardiac fibrosis [70]. miR-320c induced suppression of TSP-4 expression could further accelerate renal fibrotic processes. Recently, it was shown that cardiomyocytes mediate anti-angiogenic effects in type 2 diabetic rats via the exosomal transfer of miR-320 into endothelial cells where its angiogenesis-related target genes (IGF-1, Hsp20 and Ets2) were down-regulated [71,72].

Further studies are necessary to confirm these results, especially as this was a cross-sectional study, and a longitudinal cohort study may provide more precise insights into disease progression. Another confounding factor might be the medication of patients as they are treated with metformin and angiotensin converting enzyme inhibitors or angiotensin receptor blockers and other anti-hypertensive drugs. Our study is focused on urinary exosomal miR-320c correlation with micro-albuminuric DN patients with a small eGFR range (screening cohort: eGFR: 32–39 ml/min/1.73m^2^; confirmation cohort: eGFR: 22–55 ml/min/1.73m^2^). Therefore, patients with low eGFR and macro-albuminuria as well patients with micro-albuminuria across a wider eGFR range showing a graded correlation of miR-320c levels with UACR across the spectrum of normo- to micro- and macro-albuminuria would further support the results. Nevertheless, this pilot study provides first insight into altered urinary exosomal miRNA profile in type II DN patients. These novel, non-invasive markers show promise as tools for the mechanistic investigation of renal pathophysiology, including disease progression, and potentially to monitor treatment effects.

## Supporting Information

S1 Fig Indirect influence on the TGF-β signaling pathway via targeting TSP-1. Ingenuity pathway analysis (http://www.ingenuity.com/). Gene names marked in red represent predicted direct target mRNAs of miR-320c.
(TIF)

S1 Table Medication information of patients included in the screening and confirmation cohort. (DOCX)
