# Supplementary figures and images for: Urinary Exosomal miRNA Signature in Type II Diabetic Nephropathy Patients
Source: PLoS One. 2016 Mar 1;11(3):e0150154. doi: 10.1371/journal.pone.0150154 (PMC4773074; doi:10.1371/journal.pone.0150154)

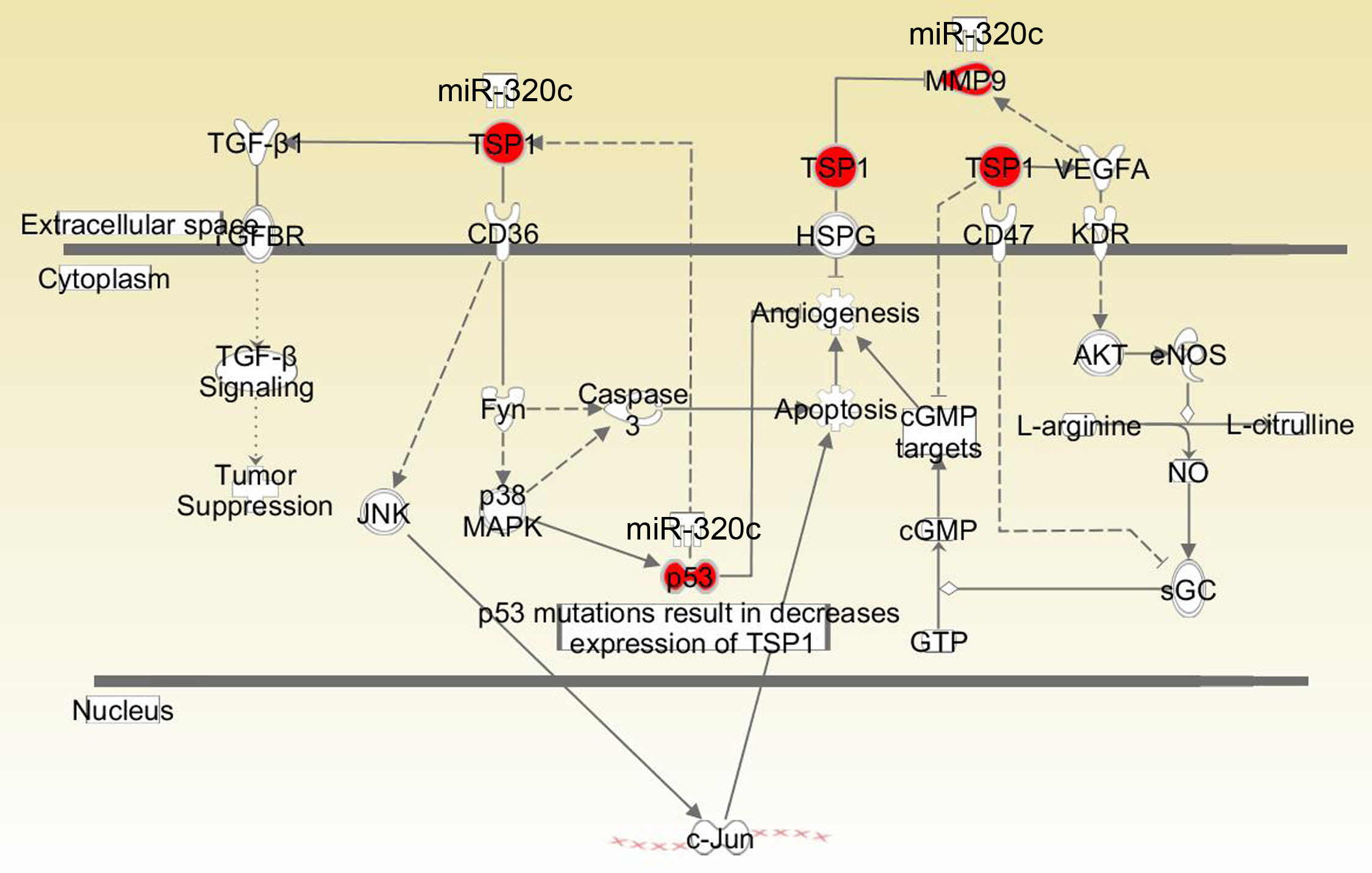

Supplement: S1 Fig — Ingenuity pathway analysis (http://www.ingenuity.com/). Gene names marked in red represent predicted direct target mRNAs of miR-320c. (TIF) [file pone.0150154.s001.tif]
