# Supplementary material for: Urinary Exosomal miRNA Signature in Type II Diabetic Nephropathy Patients
Source: PLoS One. 2016 Mar 1;11(3):e0150154. doi: 10.1371/journal.pone.0150154 (PMC4773074; doi:10.1371/journal.pone.0150154)
Supplement: S1 Table — (DOCX) [file pone.0150154.s002.docx]

|  | **Subject ID** | **ACE inhibitors** | **ARBs** | **Insulins** | **Metformin** | **Other OADs** | **Statins** | **Calcium channel blockers** | **Diuretics** | **Other antihypertensives** | **Antithrombotics** |
| --- | --- | --- | --- | --- | --- | --- | --- | --- | --- | --- | --- |
| Screening cohort | DN1 |  | x | x |  |  |  |  |  |  |  |
|  | DN2 |  | x | x |  |  |  |  |  |  |  |
|  | DN3 | x |  | x |  |  |  |  |  |  |  |
|  | DN4 |  |  |  | x |  |  | x |  |  |  |
|  | DN5 | x |  |  | x |  |  |  |  |  |  |
|  | DN6 | x |  |  | x |  |  |  |  |  |  |
|  | DN7 | x |  |  | x |  |  | x |  |  |  |
|  | DN8 |  |  | x | x |  |  |  |  |  |  |
|  | D1 |  |  |  |  |  | x |  |  |  |  |
|  | D2 |  |  |  | x | x |  | x |  |  |  |
|  | D3 |  | x |  |  |  |  |  |  |  |  |
|  | D4 |  |  |  |  | x | x |  |  | x | x |
|  | D5 |  |  |  | x | x |  |  |  |  |  |
|  | D6 |  |  |  |  |  |  |  |  |  |  |
|  | D7 | x |  |  | x | x |  |  |  |  | x |
|  | D8 |  |  |  |  | x |  |  |  |  |  |
|  |  |  |  |  |  |  |  |  |  |  |  |
| Confirmation cohort | DN1 | x |  | x | x | x |  |  |  |  |  |
|  | DN2 | x |  | x | x |  |  |  |  |  |  |
|  | DN3 |  | x |  |  | x | x | x | x | x | x |
|  | DN4 |  |  | x | x |  | x |  | x |  |  |
|  | DN5 | x |  | x |  | x | x |  | x |  | x |
|  | DN6 | x |  | x |  | x | x |  | x |  | x |
|  | D1 | x |  |  |  |  |  |  |  |  | x |
|  | D2 |  |  | x |  | x |  | x |  | x |  |
|  | D3 |  |  |  |  |  | x |  | x |  |  |
|  | D4 |  |  |  | x |  | x | x |  |  | x |
|  | D5 |  |  |  |  |  |  |  |  |  |  |
|  | D6 |  | x |  |  | x | x |  |  |  |  |
